# Access to Essential Medicines in Pakistan: Policy and Health Systems Research Concerns

**DOI:** 10.1371/journal.pone.0063515

**Published:** 2013-05-22

**Authors:** Shehla Zaidi, Maryam Bigdeli, Noureen Aleem, Arash Rashidian

**Affiliations:** 1 Department of Community Health Sciences and Women & Child Health Division, Aga Khan University, Karachi, Pakistan; 2 Alliance for Health Policy and Systems Research, World Health Organization, Geneva, Switzerland; 3 School of Public Health, Knowledge Utilization Research Center, Tehran University of Medical Sciences, Tehran, Iran; Boston Children’s Hospital, United States of America

## Abstract

**Introduction:**

Inadequate access to essential medicines is a common issue within developing countries. Policy response is constrained, amongst other factors, by a dearth of in-depth country level evidence. We share here i) gaps related to access to essential medicine in Pakistan; and ii) prioritization of emerging policy and research concerns.

**Methods:**

An exploratory research was carried out using a health systems perspective and applying the WHO Framework for Equitable Access to Essential Medicine. Methods involved key informant interviews with policy makers, providers, industry, NGOs, experts and development partners, review of published and grey literature, and consultative prioritization in stakeholder’s Roundtable.

**Findings:**

A synthesis of evidence found major gaps in essential medicine access in Pakistan driven by weaknesses in the health care system as well as weak pharmaceutical regulation. 7 major policy concerns and 11 emerging research concerns were identified through consultative Roundtable. These related to weaknesses in medicine registration and quality assurance systems, unclear and counterproductive pricing policies, irrational prescribing and sub-optimal drug availability. Available research, both locally and globally, fails to target most of the identified policy concerns, tending to concentrate on irrational prescriptions. It overlooks trans-disciplinary areas of policy effectiveness surveillance, consumer behavior, operational pilots and pricing interventions review.

**Conclusion:**

Experience from Pakistan shows that policy concerns related to essential medicine access need integrated responses across various components of the health systems, are poorly addressed by existing evidence, and require an expanded health systems research agenda.

## Introduction

Essential medicines, as defined by World Health Organization (WHO), are those that satisfy the health care needs of majority of the population. Support for access to essential medicines is pledged under Millennium Development Goal 8 and the provision of affordable, high quality and appropriate essential medicines is a component of functioning health systems [1]. However access to essential medicines in low and middle income countries (LMICs) remains questionable [2]. Cohesive evidence is essential to understanding, planning, monitoring and evaluating access to medicines [3].

There are a number of gaps related to evidence on access to essential medicines. First, although reasonably sufficient information from the Organization for Economic Co-operation and Development (OECD) countries is available on essential medicine access, the data from LMICs is often weak, fragmented and requires collation [2]. Second, even where published research on medicines is available in LMICs the evidence has usually not been well integrated the within wider health systems responses and the pharmaceutical and health systems stakeholders continue to function in silos. A health systems perspective applying health policy and system research frameworks (HPSR) is hence needed in the generation of evidence on access to essential medicines [4]. Third, ideally, such country case studies need to go beyond empirical data collection to also include consultation of local stakeholders in the generation of prioritized policy and research concerns. Prioritization of policy and research areas has been typically driven from the global level and more recently there has been a call for country level iterative priority setting exercises involving a range of stakeholders, so as to come up with more context specific and nationally driven policy and research concerns for improved health systems [5].

This paper attempts to add to the global evidence on access to essential medicines by sharing findings from Pakistan. It applies both a health systems perspective and a local priority setting exercise. The paper sets out to i) identify policy concerns in access to medicines through desk review and key informant interviews; and ii) present consultatively prioritized policy and research concerns. The results are intended to improve the use of evidence in medicines policies and forging integrated responses to related challenges within the heath systems.

### Setting

Pakistan has a population of 185 million, a Gross National Product (GNP) per capita of $1200 and a literacy rate of 53 percent [6]. Pakistan has a mixed health care system with the public sector providing services to 22 percent of population and a dominant private sector, mainly comprising of private for profit practitioners and health facilities, serving the rest of the population. The Drug Control Organization located until recently within the federal Ministry of Health (MOH) has been responsible for producer licensing, drug testing, drug registration, pricing and trade, while Drug Quality Control Boards located within the provincial Departments of Health (DOH) are responsible for market surveillance. Under a constitutional amendment in the MOH along with a number of other social sector ministries was devolved in 2011 to the provinces and the re-organization of drug regulation is unclear. The Pakistan Medical and Dental Council and the Pakistan Pharmacy Council are responsible for licensing medical and pharmacy schools and practitioners. The Pakistan Medical Association and the Pakistan Pharmacists Association represent the interests of the two main provider groups. The Pakistan Pharma Bureau represents the local industry and forms an active interface for dealings with the government on drug production, pricing and trade.

Access to essential medicines, as part of the fulfillment of the right, to health is recognized in the national constitution. Pakistan has fairly well developed policy acts and operative guidelines. The Drug Act 1976 regulates the pharmaceutical sector setting out extensive stipulations for industry licensing, drug registration and quality control. The Drug Act of Pakistan has neither been updated with the international the World Trade Organization’s (WTO) statutes nor with local stipulations such as the Pakistan’s Patent Ordinance of 2000. A National Medicines Policy was developed in 1993 and again in 1997, but do not have a strategic plan for implementation.

## Methods

### Ethics Statement

Ethical approval of the Aga Khan University Ethics Review Committee was obtained prior to start of the study. Written informed consent was obtained from each interviewee. Confidentiality of identity was maintained in the analysis and write-up by replacing interviewee identity with a code.

### Approach and Framework

**Figure 1:**
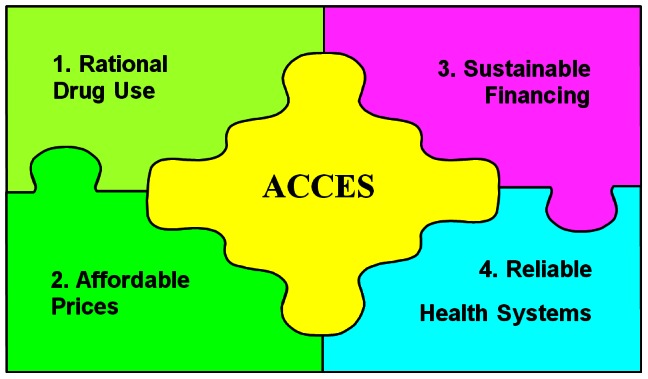
Improving Access to Essential Medicines: A Framework for Collective Action.

The Pakistan Access to Medicines (ATM) priority setting study was part of a larger global study involving 17 countries over five regions and formed the Easter Mediterranean Regional (EMR) sub-set together with Iran and Lebanon. An exploratory policy analysis was conducted using the WHO Framework for Equitable Access to Essential Medicine [7] as the conceptual basis for data collection and synthesis (Figure 1). Under this framework accessibility has been defined as having four parameters: that i) there are reliable health systems for ensuring medicines are available and effective, ii) affordable pricing, iii) sufficient health financing to remove financial barriers for patients, and iv) that required knowledge and guidance are available for rational use of these medicines. It assumes that isolated efforts to improve one aspect might not ensure adequacy of access to essential medicines. We gathered data from desk reviews and stakeholder interviews followed by a stakeholder roundtable to prioritize the emerging policy and research concerns. The desk review and fieldwork was carried out during January to May 2011 and was completed just before the devolution of the Ministry of Health in June 2011.

### Desk Review

The desk review was conducted to collate primary research and policy measures. Electronic database search of peer reviewed and grey literature was conducted by 2 researchers using Medical Subject Headings (MESH) terms (Table 1), and complemented by a hand search of bibliography. Primary research and reviews were included while commentaries and bio-efficacy studies were excluded. Study design filters were not applied to the primary research reviewed as the purpose was to obtain an overview of evidence rather than conduction of a systematic review. The MESH words used singly and in combination yielded 2176 titles that were further sifted to shortlist appropriate abstracts for review. A total of 184 abstracts were retrieved and reviewed, and shortlisted to 68 full text studies. The search was conducted by 2 researchers with Researcher 1 conducting the initial screening of titles and abstracts, while Researcher 2 reviewed the title and abstracts search, and consensus was reached between the two researchers on selection of relevant articles. All selected articles were independently reviewed by both the researchers. In addition, 19 policy documents were also identified, 14 through the online search and 5 during the course of stakeholder interviews. Information was extracted into thematic grids and organized under the four domains on the WHO Equitable Access to Essential Medicines Framework. Researcher 1 did the initial extraction, while Researcher 2 reviewed the extraction, and any disagreement was resolved through combined review of the article by both researchers and arriving at a consensus.

**Table 1:** Overview of Methods.

Desk Review		
**Online search:** Electronic databases searched:PubMed, Cochrane, Cinahal, WHOLIS, ELDIS,Google Scholar. Websites searched: Ministryof Health, Provincial Departments ofHealth, WHO Pakistan, WHO-EMRO and PakistanConsumer Protection Network.	**Search Terms:** Rationale Drug Use AND Pakistan;Drug Financing AND Pakistan; Drug AffordabilityAND Pakistan; Drug Access AND Pakistan;Drug Supply AND Pakistan; Drug AvailabilityAND Pakistan; Drug Policy And Pakistan; Pharmaceutical Policy AND Pakistan Searches conducted during Jan-March 2011 and updated in August 2012.	**Research Inclusion Criteria:** Primary research studies, reviews, case reports. Excluded: opinion pieces, commentary articles, bio-efficacy studies.
		**Grey Literature:** Policy Acts, Policy Guidelines, Policy or strategic frameworks, national formulary, documents on official mandate of stakeholders.
**Key Informant Interviews**		
**Ministry of Health:** Federal DirectorateHealthLicensing & Registration Board	**Departments of Health** Secretariat; TertiaryHospital; District Health Officer	**Private Providers:** National NGO International NGO
**Dev Partners:** WHO, GAVI	**Industry:** Pakistan PharmaceuticalManufacturing Association	**Pharmacies:** Pakistan Pharmacists Association Hospital Pharmacists Society
**Researchers:** Bio-Equivalence Centre, NationalHealth Systems Strengthening Unit	**Advocates:** Consumer Protection Network	**Clinicians:** Pakistan Medical Association
**Roundtable**		
**21 Participants:** Representatives from Ministry ofhealth, provincial Health Departments, District HealthOffice, Presidents Primary Health Care Initiative, PakistanPharmaceutical Manufacturing Association, PakistanMedical Association, Pakistan Pharmacists Association,WHO, pharma experts, heath system experts	**Sequence of Activities:** Presentation of findingsPresentation of emerging concerns Validation/correction of information through verbal adhoc andwritten participant pot hoc feedback Commentsby participants on findings	Group discussion on policy concerns and prioritization Group discussion on emerging research concerns and priortization

### Key Informant Interviews

Through consultation amongst the regional study teams of Pakistan, Iran and Lebanon a listing of stakeholder categories was developed so as to get representation from diverse stakeholders and the listing comprised of the Ministry of Heath, Departments of Health, Industry, Researchers, Development Partners, Advocates, Clinicians, Pharmacists and Private Providers (Table 1). Stakeholders within each listed category were identified by the country team with assistance of the WHO country office, and further additions to the list made through a snowballing approach. A total of 21 interviews were conducted across the different stakeholder categories to collect views on key issues related to access to medicines in Pakistan and need for evidence (Table 1). Semi-structured interviews were guided by a topic checklist, one researcher conducted the interview while the other took notes, and notes were manually transcribed soon after the interview. Interviews notes were finalized after review and consensus by both researchers. The transcripts were manually coded by Researcher 1 by organizing emerging issues under conceptual framework themes, and the initial coding was reviewed and finalized by Researcher 2.

### Stakeholders’ Roundtable

Findings of interviews and desk review were consolidated under the domains of the ATM conceptual framework and shared in a Roundtable with the stakeholders (Table 1). The purpose of the Roundtable was to share preliminary findings for validation, hear further from the stakeholders on policy and research concerns and undertake a consultative prioritization of the concerns highlighted by the fieldwork and desk review. The Roundtable was attended by 21 participants drawn from the broad range of stakeholders interviewed (Table 1), the regional partners and WHO Alliance Health Policy and Systems Research (HPSR).The Roundtable was jointly moderated by Researcher 2 and a WHO representative, and note taking done by Researcher 1 and supplemented by Researcher 2. Validation of collected data was undertaken with the participants reviewing the information during the Roundtable meeting, updating information in certain areas, and pointing out supplementary data sources. Written post hoc comments on data validity were also invited through an email list serve and incorporated. A focus group methodology was applied for collective prioritization as participants opted for a consensus building discussion to prioritize rather than quantitative scoring on Likert scale. A moderated discussion was conducted first involving commenting on findings by each participant followed by group discussion on prioritization of concerns. Ranking was therefore not attempted and instead an agreed list of concerns was developed.

## Results

### Desk Review

A total of 72 documents were reviewed that included 53 grey and published studies, and 19 policy related acts, stipulations and guidelines. English was the main language of publication and locally produced evidence was the major source with 47 out of 53 publications from Pakistan. However studies having nationally representative samples were few. Highest number of literature related to rational drug use (27) with least on medicine financing (3).

#### Rational Drug Use: Much known but little action

Evidence generated by desk review largely related to rational drug with markedly much less on other domains. Within the rational drug use area, the major volume of research related to prescribing practices of health care providers, there were few studies on dispensing and community pharmacy, and no research related to drug regulation policies and on consumer related factors. Studies largely did not follow standardized methodologies and lacked nationally representative samples.

Pakistan has an Essential Drug List (EDL) currently containing 335 medicines [8] and is complied by 80 percent of public sector facilities [9]. Despite the existence of an EDL medicines have been registered in excessive numbers comprising 1100–1200 registered molecules and 50,000 registered drug products. Registration does not look into comparative cost analysis over other products and local bio-equivalency is not required [10]. The average number of drugs prescribed per patient in Pakistan is over 3 compared to an average of 2–3 in LMICs [2], with higher prescription of 4.5 in private sector prescriptions [11], compared to 2.77 in public sector [12]. Injection use is excessive with 60 percent of patient encounters involving an injection [2]. Antibiotics use is also high, more so amongst privately practicing general practitioners with 62 percent of prescriptions involving antibiotics versus 54 percent in public sector [13]. Little difference exists between privately practicing general practitioners and specialists in terms of excessive antibiotic use [14]. Apart from poly-pharmacy, prescription patterns are also inappropriate even for frontline health problems such as tuberculosis [15], childhood diarrhea [14], acute respiratory infection [16], hypertension [17], diabetes and anxiety/depression [18]. Interaction of health providers with the industry is not restricted and visit of sales representatives is linked with increased prescription of the sponsored medications [19]. Drug dispensing time in the public sector is insufficient for patient instruction and does not adhere to standard safety measures for dispensing [12], however comparable figures are not available for the private sector. Community pharmacy is also weak with little restriction on over-the-counter medicine purchase by patients and sub-optimal quality standards are followed with only 12 percent of drug retail outlets having pharmacologically trained dispensers [20] and only 19.3 percent meeting licensing requirements [21]. Self-medication of antibiotics even two decades ago ranged between 6–8 percent in the general population [22]–[23] with updated evidence likely to show higher self-use.

#### Affordability: Pro-poor measures but low trickle down

The national affordability and pricing survey conducted in 2006 [24], using WHO standardized methods provides comprehensive information on pricing in both the public and private sectors. Apart from this there is little research on pricing, and no attempts at periodic updating of information.

Pro-poor measures are consciously maintained by the MOH involving tax exemption on imported raw material and equipment for drug manufacturing, exemption of drugs from general sales tax and full tariff exemptions on drugs imported by United Nations (UN) agencies and donor funded programs [25].The Drug Act 1976 is vague about pricing, and pricing is decided on case by case basis and based largely on input cost [26]. Drug affordability despite pricing measures continues to be a concern in Pakistan mainly due to proliferation of originator brands and wide price variability. Availability of basket of essential generic medicines is low in public sector (15%) and sub-optimal even in private sector (31%) [24].The price ratio of branded products to international reference price ranges between 0.72 to 26.2 showing excessive price variability while the corresponding ratio for generics is between 0.2 and 7.02 [24].Specific medicines such as omeprazole, ciprofloxacin and diclofenac suffer from excessive prices. Poor drug availability in the public sector forces patients to purchase from private retail outlets, as further discussed in the next section. Affordability index as defined by WHO is more than 1 day’s income by lowest paid government worker for 1 month’s standard treatment of chronic illness or for one episode of acute illness [27]. While acute therapy using generic was found to be affordable for acute respiratory infection at 0.3–1 days wage, therapy for chronic illness such as hypertension, depression, diabetes, epilepsy, arthritis and peptic ulcer is unaffordable even with use of low priced generics at 1.7–7.7 days wage and clearly beyond reach of poor with originator brands at 1.9–36.4 days wage [28].

#### Sustainable Financing: a case of low funding or inefficient management?

In Pakistan nationally representative data is available on medicine expenditure by the public and private sectors from of the National Health Accounts. There are also random studies on patient expenditure at specific health care facilities. However, regular surveillance of medicine expenditure is not being done although required for monitoring the results of policy changes and of health systems innovations.

Pakistan has a total spending of USD14 per capita per year, much below the USD 34 recommended by WHO for developing countries, and the public sector constitutes merely 32 percent of total health expenditure with 64 percent borne by households mainly through out of pocket payments [29]. Medicines account for a substantial 43 percent of total household health expenditure in Pakistan [29]. Within the public sector only 22 percent of operational budget is available for non-salary items including drugs [30]. The amount expended for drugs in public sector is below the critical threshold of $2 per capita per year recommended by the WHO to avoid medicines shortages [31]. Evidence indicates substantial hidden cost of medicines at public sector facilities as patients due to low drug availability are often forced to purchase from private retail pharmacies. Mean out-of-pocket spending per prescription is Rs.252 at private sector facilities compared to Rs198 at public sector facilities [32].

Although experimentation with new health delivery and financing schemes has been initiated in Pakistan involving vouchers pilots and an extensive national contracting-in initiative at the primary care level [33], reduction in drug expenditure is yet to be ascertained. Zakat funds - religious welfare tax for use of Muslims –accounts for 1 percent of total health expenditure and are expended on drug purchase for poor patients at public sector hospitals [29], but there has been no assessment of Zakat fund utilization. Private philanthropies contribute towards the cost of drugs at public sector tertiary hospitals, but these are concentrated in urban areas, are fragmented, and have not been evaluated for medicine access [34].

#### Reliable Health Systems: missing the policy spotlight

Health systems are expected to ensure sufficient production, quality assurance, adequate supply management of essential medicines and appropriate human resources. Published evidence in this area is scarce, the few studies available mainly report on drug availability in the public sector, with little primary research in the areas of drug procurement, logistics management, quality assurance and sufficient production. The data sources for these areas are mainly drawn from government records.

There has been a stride in drug production since the country’s Independence in 1947 with currently 30 multinational and 411 local manufacturing units [25]. However self sufficiency is yet to be achieved with only 35 percent of domestic demand met by local manufacturing units [35] and raw material for local drug production is almost entirely imported. Quality assurance mechanisms for licensing of manufacturer licensing, drug product registration and market surveillance are well laid out by (Drug Act 1976) however the profusion of drug production outlets and drug products raises questions about the tightness of controls. The issuance of Statutory Regulatory Orders reportedly creates confusion and unevenness in the application of policies [36]. At present in Pakistan none of the manufacturing facilities are WHO certified. Market surveillance conducted by the provincial Departments of Health involves sampling of drugs on the market but there still continues to be a high proportion of counterfeit drugs [26], [37]. Surveillance is restricted to drug product sampling and overlooks quality parameters of retail outlets (Drug Act 1976).

There are frequent stock-outs of essential drugs across primary, secondary facilities and district hospitals (34). Contracting out the management of frontline facilities in selected districts has improved drug availability with 22.5 percent of contracted facilities in the highly satisfactory category for drug availability as compared to 8.3 percent of non contracted facilities [38]. Drug availability has improved in disaster affected areas where drug distribution was managed through a network of UN agencies and international NGOs and was linked with better inventory control and computerized logistics support [39]. Drug storage in public sector does not follow standard operating procedures. A survey of public sector facilities found that the manual for procedures was available in only 5 percent of public sector facilities, refrigerators were working in 60 percent and temperature control was present in 24 percent [9]. Supply management in the private health sector is also sub-optimal with available evidence indicating that only 50 percent of private facilities comply with the national EDL [11] and merely 19 percent of drug retail outlets meet licensing requirements [21]. Evidence for private sector is confined to small scaled studies and needs national representative surveys.

Pharmacist availability is low across public and private sector with only 0.06 pharmacists available per 10000 population, much below the recommended ratio of 5 pharmacist per 10000 population [25]. Majority of pharmacists work in pharmaceutical industry (70%), with the rest distributed over hospital pharmacy, community pharmacy and academia [40].

### Key Informant Interviews

Key informant mainly identified policy concerns as perceived by them, with only a few respondents belonging to experts and development partners additionally identifying research concerns. One reason was that several of the policy concerns called for policy actions rather than research. Another reason was that the range of informants interviewed was not well familiar with policy related research.

#### Irrational Use

*“Essential generics are the gold standard but the problem for prescribers is they have been around in the market for long time, they don’t have star status like new brand drugs, they don’t create awe in the market.” (Interview: 21/21).*

Irrational drug use was thought by informants to be both widely prevalent as well as the most complex issue. Informants stated that prescribing practices need improvement from specialists to general practitioners, and unauthorized prescriptions by quacks requires regulation. Informants thought that even amongst well meaning practitioners, generics having been around for a long time did not enjoy the same prestige as new brand products. Frequent shortages of low cost generics in the market further strengthen use of irrational branded drugs. Open access of doctors to industry representatives, lack of refresher training, demand for quick cures by patients and entrenched parallel quackery were felt to sustain irrational prescriptions. Similarly, little restriction over self-medication by patients, lack of pharmacist presence at drug retail outlets and low levels of patient awareness were felt to further enforce irrational use.

*“Rational use is one of the biggest barriers in access to medicine. There are no qualified pharmacists at the pharmacy. Then the role of marketing by pharmaceutical companies is not ethical whereby doctors are attending conferences in Dubai and prescribing expensive medicines and getting benefits from these companies.” (Interview4/21).*

At the program level, the domination of procurement by clinical specialists, little institutional role of pharmacists in supply management and weak enforcement of Essential Drug Lists and available standard treatment protocols, emerged as the major contributory factors.

*At every level there should be implementation of a formulary. Institutions should be bound to use that. Second step is protocol development. Changing attitude of senior doctors for rational prescription and avoiding poly pharmacy is very important.”(Interview 18/21).*

Another strong concern was an absence of tight policy levels controls thereby resulting in excessive registration of drugs. Stakeholders pointed to lukewarm political support for regulation of the private sector, as evidenced by an anti-quackery bill that had been drafted some years ago but was yet to be legislated, and little movement by the medical community on restricting industry access to health providers. Informants called for a multi-pronged strategy addressing policy to consumer levels for controlling irrational drug use.

#### Affordability and Pricing

*“Manufacturers of drugs don’t find it financially viable to produce thyroxine, but they prefer to make ciproxin. Hydrochlothiazide, folic acid and primaquin and magnesium sulphate are even not available in many of the private and public facilities.” (Interview:1/21).*

All key informants expressed concerns related to drug affordability and pricing issues. Respondents pointed to a steady proliferation of expensive originator brands at little additional value. For example seven different forms and prices of Acetaminophen currently existed in the market. Although the Generic Drug Act was introduced in 1972 it had to be revoked in the wake of strong opposition by the commercial sector and the medical community. Another concern was an absence of a clear pricing formula as the existing pricing practice was based on reported price of inputs. This resulted in wide price variability and was thought to also create opportunities for collusion to obtain high prices.

*“Biggest barrier to access to medicines is at the level of affordability, and my recommendation is that Generic system of medicines should be introduced in the country and pricing tag should be of MoH to considerably overcome issue of affordability particularly among white collared people and poor.”(Interview: 18/21).*

Weak regulation of distribution and sale of drugs was thought to push up the prices and the authority of Drug Inspectors to control monitor prices was also apprehended to be weak. Yet another concern was the flat price control in place on essential drugs since nearly last ten years which had counter productively resulted in the disappearance of low cost essential generics from the market due to lack of a profit margin. Declining profits due to rising inflationary costs was cited by the industry as one reason for low interest in manufacturer of low priced essential drugs however the Ministry has been reluctant to lift the price freeze due to fear of steep increase in prices and anticipated political fallout of inflationary medicine prices. The list of such ‘orphan drugs’ reported was alarming and included basic essentials such as phenytoin, thiazides, adrenaline, thyroxine, primaquin, and folic acid amongst others. The MOH’s response has been to enforce the production of ‘orphan drugs’, which in turn has triggered sub-standard production of essential medicines. So far differential pricing measures have not been explored. In contrast non-essential medicines have had periodic across the board increases and do not face market shortages.

#### Financing

*“Spending is low…more precisely, proper utilizations of funds and rationalization of drugs are not done, which are mainly driven by personal interests” (Interview: 2∶21).*

Stakeholders expressed lesser concern over adequacy of drug financing as compared to other domains of the framework. Opinion on drug financing was divided as to whether poor availability of drugs in public sector is due to under-funding or inefficient budget management.

Although public sector drug procurement involved generic purchasing and was supposed to be efficient, however stakeholders expressed concern on the frequent deviation from essential drug list and replacement of generics with originator brands particularly at hospitals which resulted in cost inefficiencies.

*“Problem is due more to lack of proper management of drug budget rather than budget shortage. There is inappropriate purchasing. Although a list of 126 drugs are approved at provincial level by Secretary Health but District Health Officer purchases from within this list as well as from their own wish list leading to inappropriate purchasing and corruption.” (Interview 3/21).*

Opportunities for collusion in procurement, drug pilferage due to weak linkage of inventory with patient consumption, and procurement de-linked to patient volume and morbidity data were other issues reported and together contributed to inefficient management of funds.

*“Procurement practices need to be improved, made more transparent and competitive.” (Interview 16/21).*

#### Reliable Health Systems

*“An open registration policy exists in Pakistan, every drug gets registered. More than 75000 drugs are registered, but there is no list (of registered) drugs with the government.”(Interview: 1/21). ‘.*

Within the area of reliable health systems the strongest concerns related to ineffective regulation of the pharmaceutical sector followed by supply management related concerns. Stakeholders pointed to the wide variety in quality of drug production units with the local market ranging from sophisticated manufacturing units having well developed quality monitoring mechanisms to low cost units having non-existent quality assurance systems. There was felt to be little incentive for producers to invest in quality control as sub-standard drugs also got registered.

*“There is little incentive to produce well, why should industry invest in quality assurance when others can get away with without such internal checks.” (Interview: 13/21).*

Market surveillance was considered to be weak and attributed to under-equipped Drug Inspectorates and their testing laboratories, and further compounded by the excessive number of drug retail outlets requiring. There were also concerns that low pay of drug inspectors and high responsibilities create opportunities for collusion with inferior suppliers and distributors. Stakeholders felt that an absence of autonomy for the drug regulatory structure was a major bottleneck for quality assurance and pointed to the Supreme Court injunction in 2005 for creation of an autonomous body which was still awaiting implementation.

*“We have always focused on macro-economic policies but attention to service delivery level; has been lacking …there lies the gap.”(Interview 5/21).*

Supply management was considered by informants to be weak but got less attention compared to regulatory concerns. Those concerned with supply management aspects felt that it commonly gets overlooked with policy spotlight usually on registration and pricing issues. Stakeholders had concerns that procurement in the public, sector despite new rules of business, favors the cheapest bids as quality parameters for drugs are low merely requiring registration of the drug production company. Moreover, stakeholders expressed concern that many of the better quality producers reportedly stay away from public sector tendering due to low priced tenders and concerns over unreliability of government as a payer. Drug storage and inventory management on the other hand have more well developed standard operating guidelines but were thought to be poorly enforced.

Low number of pharmacists in service delivery was a common concern with most stakeholders, pointing to the meager numbers even within large teaching hospitals, as for example only 1 pharmacist was posted in the largest Civil Hospital at Karachi with an OPD of 800 patients/day and 1500 beds. Stakeholders called for effective institutionalization of hospital and community pharmacy with stronger emphasis by experts and pharmacists on pharmacists’ roles in supply management as opposed to the medical community.

Although not included in list of topics, the devolution of the Health Ministry, was an area brought up by almost all informants. Most stakeholders favored some role of the federal level in standardizing drug licensing, registration, pricing and trade but with allowance for increased feedback of provinces, experts, industry and other stakeholders. Total devolution was largely felt to create inequities in terms of drug pricing, availability and quality across the provinces. There was concern expressed by federal stakeholders and experts of uneven provincial capacity for undertaking drug regulation while provincial governments felt that inclusion of provincial voice in accountability is the major issue to be addressed.

*“We are asking for a Drug Registration Authority at the federal level for registry, pricing and trade, to be kept even after devolution of the Ministry (of Health) to the provinces. We cannot have a drug registered in one province and de registered in another, or charging different prices in two different provinces.” (Interview 13/21).*

### Roundtable Discussion: Prioritization of Policy Concerns and Research Areas

Seventeen policy and 12 research concerns were shared in the roundtable based on key areas emerging from the interviews and desk review (Tables 2 & 3). These were prioritized and reduced to16 policy concerns and 7 research concerns (Table 4) through a moderated discussion, as described under Methods. Participants agreed that access to medicines is a major issue in Pakistan and majority of the stakeholders identified with reliable health systems as the major area to be addressed followed by pricing and then rational drug use. Opinion was divided over the extent of work undertaken in this area with some expressing that substantial policy work had been undertaken but met with varying success while others thought that a significant policy attempt is yet to be made.

**Table 2:** Identified Policy Concerns from Desk Review and Key Informant Interviews.

	Policy	Program	Service Provider	Consumer
**Irrational Use**	Excessive registration of drugs	Procurement dominated by clinicians in public and private sectors Purchase of originator brands in private sector	Inappropriate prescriptions Absence of standard protocolsExcessive injection use No checks at interaction with industry	Patient demand for quick cures Low awareness Few restrictions on over the counter access
**Pro-poor policies but low affordability**	Flat price control Proliferationof originator brands athigh prices Unclear pricingformula	Market shortages of essential low cost generics	Preference for prescribing originator brandSwitch from generic use to originator brand triggered bymarket shortages	Acute illness therapy affordable at only generic prices NCD therapy unaffordable at even generic prices
**Insufficient Financing for drugs**	Insufficient public sectorspending on health	Health budgets dominated by salaries Health equity funds: sporadic and unmonitored useLack of alternative financing models having drug subsidies	Drug stock-outs in public sector Improved drug availability with contracting out but questionable quality Prescription of originator brands in private sector	Highest OOP share spent on medicines OOP on medicines incurred at both private and public sector
**Weak Health Systems**	Drug production reliance onboth local and multinationalcompaniesLow quality thresholdfor drug registration Fragmentedmandate for pharma policy acrossfederal and provincial levels in postdevolution context	Counterfeit medicines but insufficient resources for market surveillance Cost efficiency but low quality in drug procurement: public sector Lack of adherence to national formulary: private sector Insufficient production and deployment of pharmacists Weak logistic management and information systems	Inadequate dispensing skillsWeakness in drug storageProliferation of shadow pharmacies	Social accountability mechanisms needed

**Table 3:** Identified Research Concerns from Desk Review and Key Informant Interviews.

Research Concerns:	
1.	Impact of decentralization on prices, availability and access
2.	Determinants underlying weak implementation of existing medicines policies
3.	Decision making role of pharmacists for medicine supply management
4.	How to improve pricing policies for better access to essential generic drugs
5.	Role of private sector particularly shadow pharmacies in drug prescription, stocking and dispensation
6.	Post-marketing assessment of drug quality
7.	Information, availability and transparency in public domain
8.	Operational research for development of a medicines information system
9.	Consumer Health seeking preferences and underlying determinants
10.	Monitoring of market medicine price to inform pricing regulations
11.	Unit cost estimation for optimal pricing of drugs
12.	Transparent information on registered drugs and prices for public consumption

**Table 4:** Prioritized Policy and Research Concerns through Stakeholders’ Roundtable.

Prioritized Policy Concerns:		Research Concerns:	
1	Too many registered products and low quality threshold for drug company registration	1	Surveillance of policy, including decentralization, on prices, availability, and quality
2	Post devolution need for independent drug regulation authority and greater voice of all stakeholders	2	Best practice lessons learnt from LMICs for pricing policies, particularly controlling availability of ‘orphan drugs’, market price variations and unit cost price estimation
3	Lack of incentives to produce quality drugs	3	Investigating the success and failures of the essential medicines programme and driving factors
4	Clear cut pricing formula not in place and decided pricing not easily available nor enforced	4	Operational pilots for improved supply management including new financing mechanisms, medicines information system, and pharmacist’s role in decision making
5	Flat price control is counter productive resulting in disappearance of low cost priced drugs	5	Mapping private licensed sector and ways to increase access through private sector
6	Burden of medicine payment mainly on households	6	Examining consumer preferences for medicine use and underlying drivers
7	Unnecessary, and often inappropriate prescriptions, by medical practitioners	7	Transparency and availability of information related to medicine use
8	Little presence of therapeutic protocols & formularies in health facilities		
9	Lack of public sharing of EDL, irregular updating and weak linkage with morbidity data		
10	Low availability of medicines in public sector at all tiers of health system but improved availability with contracting –why?		
11	Inadequate operational budget for medicine in public sector and existing budget needs to be more efficiently managed		
12	Need for centralized procurement in public sector and quality checks		
13	Outdated logistics management systems		
14	Weak hospital pharmacy across public and private sector		
15	Proliferation of shadow pharmacies		
16	Large and unregulated private sector and popularity of informal providers and quacks		

Although ranking was not attempted, weak regulation related to drug registration and market quality surveillance emerged as the most critical policy concern amongst participants with a call for better implementation of existing regulations and tightening these in needed areas (Table 5). Next, were policy concerns related to drug pricing and provider prescribing practices, with calls to make more transparent pricing formula, creative pricing policy to counter drug shortages, and multi-pronged action for irrational drug use. This was followed by supply side concerns ranging from budget insufficiency for drugs, procurement, storage and dispensation gaps, to low deployment of pharmacists. Lack of community level actions was raised as a concern by some participants and there was agreement on a need for better accountability mechanisms. Impact on essential medicines of decentralization of health to provinces, was additionally introduced by participants as a common concern. While participants had different views on desirability of devolution, a consensus was reached that while a central structure is needed to avoid inequitable drug availability and pricing across provinces, there needs to be autonomous functioning and greater participation of provincial government as well as other stakeholders. The concerns expressed in the roundtable generally conformed to the trend reported in the key informant interviews.

**Table 5:** Prioritization of Policy & Research Concerns: Stakeholders’ Perceptions.

*Dissemination and transparency of information in the fields of registration and licensing of medicines is the need of hour in Pakistan. The Ministry of Health needs to provide publically list of registered and deregistered drugs.” (Stakeholder 2/21).*
*“Optimal mix of pricing regulations is needed to reduce expenditure burden on households. Moreover continuous surveillance of impact of policies on availability, price and affordability is needed.” (Stakeholder 6/21).*
*“As far as prescribing is concerned, both private and public sector should follow the Essential Drug List. And there needs to be strict regulation and monitoring for it.” (Stakeholder 15/21).*
*“Availability of essential medicines are compromised because of prescriptions written by the general physicians and specialists working in the private sector. For instance, if you observe clinics in Lahore, Faisalabad, these (private) doctors have made pharmacies inside their clinics and prescribe only those drugs which are available with them (Stakeholder 9/21).*

Based on the prioritized policy concerns, participants identified a range of research priorities. A need was identified for continuous market and public sector surveillance to look into the effect of national policies on medicine availability, prices and quality. Other mentioned research concerns were the need for standardised prescription and dispensing audits of health providers and development of a database of private licensed providers differentiating these from unlicensed and informal providers. Participants also recommended the collation of best practice lessons on pricing policies so as to improve access to essential generics. The need for financing research pilots also emerged to find innovative means for reducing medicine expenditure borne by households particularly for chronic care therapy. Operations research for improving logistics management in the public sector was also proposed applying best practice lessons from credible NGO managed models. Another research priority was the call for formative research to look into consumer demand, health-seeking preferences, willingness to pay, and enhancing patient role in accountability.

## Discussion

The essential medicines area has been under-explored in health systems research and evidence is particularly thin on country level contextual findings from LMICs. Furthermore country level perspective in identifying policy and research concerns has been less well incorporated in country case studies, with medicine policy and research driven by global priorities. We share findings from Pakistan reporting key challenges in access to essential medicines supplemented with a nationally driven prioritization of policy and research concerns.

A synthesis of evidence found major gaps in essential medicine access in Pakistan related to weak regulation of quality assurance, poor affordability, and irrational use. These are driven both by weaknesses in pharmaceutical regulation with little attention to quality and cost efficiency in drug registration and a lack of creative and transparent pricing, as well as by health systems weaknesses involving unregulated provider prescriptions and weak supply management.

These require cohesive policy responses involving the provision of autonomy and capacity for drug regulation, inclusion of safety nets such as health insurance for affordable medicine financing, private provider regulation and community pharmacy to curtail excessive prescription, and use of more methodical procurement practices.

Similar priority setting exercises have been recently conducted in 16 other LMICs. Pakistan in contrast to other countries has higher concerns related to drug quality regulation and reliable health systems, but shares medicine pricing and rational use concerns with a number of other countries. Affordability and financing dominate in the EMR, India, Vietnam and Ghana [41]–[44]. Rwanda, Cameroon, Gabon, Chad and the Congo highlight the impact of payment mechanisms on access to medicines [45], [46] and socio-cultural factors affecting access as the major concerns [46]. The Latin American Countries mention quality assurance as an issue as well as high cost medicines and cost-containment policies [47]. Regulation and community services are concerns raised in Lao PDR [48]. Many countries also worry about access to medicines for specific populations and disease conditions, especially chronic non communicable conditions.

We found there was a mismatch between the available research and identified policy concerns. Most research tended to be on irrational prescribing with fewer studies related to the policy concerns on regulation, pricing and supply management. The Pakistan experience highlighted the need for trans-disciplinary research to address identified policy concerns. Areas of critical need are surveillance studies of drug availability, pricing, quality and use, consumer behavioral research, interventional pilots related to drug financing and supply management, and systematic reviews of pricing interventions. There are similar gaps related to access to medicine research in other LMICs. Medicine pricing and availability surveys have been undertaken in a number of countries [27] with WHO support but require periodic conduction in all countries and use of standardized parameters. Interventional research on drug access and use in developing countries is also extremely limited in developing countries as also seen in Pakistan [49], [50]. In-depth understanding of consumer behavior and experiences in relation to medicine use is another common research gap in developing countries as is the case in Pakistan.

This study has three main strengths. First, the study provides context relevant evidence from a developing country setting given that access to essential medicine is a common issue across developing countries and faces a dearth of evidence. Second, it applies a health systems lens to essential medicine access which offers the advantage of building important interconnections across systems components to avoid fragmented, vertical and narrow commodity based solutions to medicines access [4]. Finally, it incorporates an iterative nationally driven prioritization of policy and research concerns. According to the Working Group on Priority Setting (2001) [51], locally driven research priorities and use of qualitative process are considered more apt for health systems priority setting rather than more quantitative close ended scales used in disease ranking [52]. Although similar country level priority setting exercises have lately been applied in the areas of human resource [52] and health financing [53] in LMICs they have not been previously applied to the area of access to essential medicines. It also includes a multiple range of stakeholders in the priority setting process as advocated for a systems perspective [54]. It also suffered from weaknesses. The desk review found studies of varying study designs, sample sizes and quality, and could have benefitted from standardized research and nationally representative samples [55]. The results may have been biased by purposive selection of stakeholders however we tried to reduce bias by including a broad range of stakeholders. Key informants had difficulty in identifying research priorities for access to medicines, and this may be due to a narrow bio-medical interpretation of research or that not all policy concerns require a research action. Similar difficulties in eliciting research concerns have been observed in priority setting exercises in other health system areas [56]. We found that the iterative roundtable process was more effective in eliciting research priorities. Finally the conceptual framework could have constrained the questions asked from key informants however this was compensated our choice of a broad and flexible framework, whereby topics not originally foreseen by the framework but raised by informants were included (e.g. the issue of devolution of services in Pakistan).

### Conclusion

Pharmaceutical policy and health policy have traditionally co-existed separately in developing countries with little effort to forge linkages. The Pakistan experience shows that policy concerns related to essential medicine access in Pakistan need integrated responses across various components of the health systems, are poorly addressed by existing evidence, and require an expanded health systems research agenda. At the same time adequate steps need to be taken to allow sustained dialogue between multiple stakeholders and a continuous culture of research feeding into evidence based policies.
